# Transition From Full Mu Opioid Agonists to Buprenorphine in Opioid Dependent Patients—A Critical Review

**DOI:** 10.3389/fphar.2021.718811

**Published:** 2021-11-23

**Authors:** Michael Soyka

**Affiliations:** Psychiatric Hospital, University of Munich, Munich, Germany

**Keywords:** opioids, opioid dependence, methadone, buprenorphine, induction, transition

## Abstract

Methadone, a full opioid agonist at the mu-, kappa-, and delta-receptor, and buprenorphine, a partial agonist at the mu receptor, are first-line medications in opioid maintenance treatment. Transition from methadone to buprenorphine may precipitate withdrawal, and no accepted algorithm for this procedure has been developed. Current treatment strategies recommend transfer from methadone to buprenorphine predominantly in patients at low doses of methadone (30–40 mg/day). There are some reports indicating that transition from higher doses of methadone may be possible. A number of dosing strategies have been proposed to soften withdrawal symptoms and facilitate transfer including use of other opioids or medications and especially microdosing techniques for buprenorphine. The case series and studies available thus far are reviewed.

## Introduction

Opioid dependence is a chronic relapsing disorder causing enourmous social and economic harm (Degenhardt et al., 2014; Neusser et al., 2020). There are about 26.8 million opioid dependent people worldwide. The years of life lost due to opioid dependence has been estimated at 3.6 million in 2016 with overdose the leading cause of death, followed by suicide, accidents, infections such as HIV and hepatitis, among others (Degenhardt et al., 2009). Heroin remains the most widely abused opioid with increasing numbers dependent on prescription opioids, especially fentanyl and oxycodon (Drew, 2019; Bell and Strang, 2020).

Opioid maintenance treatment (OMT) is the established first-line treatment for opioid dependence (Amato et al., 2011; Mattick et al., 2014; Jordan et al., 2019; Bell and Strang, 2020). The evidence base for beneficial effects of OMT on mortality rate, morbidity, psychosocial functioning, criminality and the well-being of opioid users has clearly been established in numerous trials and longitudinal follow-up studies (Hser et al., 2014; Mattick et al., 2014; Hser et al., 2017; Evans et al., 2019; Bell and Strang, 2020). Unfortunately there is also evidence that mortality risk in OMT remains high, especially during the first 4 weeks of treatment or after treatment cessation (Cornish et al., 2010; Cousins et al., 2011; Kimber et al., 2015; Sordo et al., 2017).

The ongoing COVID pandemic is a therapeutic challenge both for physicians and patients with opioid use disorder. Methadone and buprenorphine are the two gold standards in opioid maintenance treatment (Amato et al., 2011; Mattick et al., 2014; Schuckit, 2016; Soyka et al., 2017). Methadone is an extensively studied medication for OMT. Methadone is a synthetic full opioid agonist with high opioid receptor binding (mu, kappa, and delta subtype), requiring daily dosing. Methadone is orally active and has a long elimination half-life. Buprenorphine has to be given sublingually because of its first pass effect and is a partial agonist with a strong binding affinity to the mu-opioid receptor and an antagonistic effect at the kappa-receptor. Buprenorphine has a weaker intrinsic activity at the mu-opioid receptor compared to methadone and a ceiling effect on respiratory depression. There are also fewer drug interactions compared with methadone. Buprenorphine has also been discussed as a medication for treatment of mood and anxiety disorders, both being frequent in opioid dependent subjects (Pendergrass et al., 2019). Whether the addition of naloxone to buprenorphine minimizes the risk for diversion or i.v., use of buprenorphine and thus providing a better safety profile is still controversial (Kelty et al., 2018). Methadone may be preferred by patients seeking sedation and or wishing to continue using opioids, while buprenorphine may be preferred by patients not seeking strong sedation and possibly heading for abstinence as indicated by a small qualitative study (Bishop et al., 2019), although defined criteria for allocation of patients to one medication or the other have not clearly been established (Crotty et al., 2020). Major advantages of buprenorphine are the lesser risk for respiratory depression, less severe withdrawal symptoms upon discontinution, and the chance of alternate-day dosing (Mattick et al., 2014; Kimber et al., 2015; Sordo et al., 2017; Soyka et al., 2017; Bell and Strang, 2020), and possibly a greater reduction of opioid use in patients with comorbid mental disorders compared to methadone (Hser et al., 2021).

In some studies the retention rate in buprenorphine patients was lower compared to methadone (Hser et al., 2014) although in general data are mixed on this issue and long-term naturalistic studies did not find differences (Soyka et al., 2017). A recent systematic review on retention rates in opioid maintenance treatment included 67 studies and found a median retention rate of 57% at 12 months (3 years: 38.4%). Drug dosing, age, other substance use (heroin, cocaine) and negative attitudes toward treatment are of relevance in this respect (O’Connor et al., 2020). Recently several long-acting buprenorphine formulations have been tested, approved, and in part introduced into clinical practice (Soyka, 2021).

Initiation of treatment resp. the induction phase is crucial in OMT, both in treatment naive patients and especially in those with change of medication. The poorer retention for buprenorphine in some trials was largely attributed to inadequate, too low dosage and discomfort in the induction phase (Amato et al., 2011). Thus, the transfer from a full opioid agonist like methadone to a partial agonist like buprenorphine poses significant challenges and is critical for further retention in buprenorphine treatment. There is broad evidence that the mortality risk is increased in patients dropping out of opioid maintenance treatment (Sordo et al., 2017). Typically, replacing a full opioid agonist like methadone by a partial agonist like buprenorphine will precipitate withdrawal in an opioid dependent patient (Strain et al., 1995; Walsh et al., 1995).

Here possible strategies in transfering patients from methadone to buprenorphine are reviewed, with emphasis on pharmacological strategies.

## Methods

To identify possible strategies and optimal tactics to transfer patients from methadone to buprenorphine a systematic Pubmed literature research was conducted, using the key words methadone and buprenorphine and transfer (9 hits) or methadone and transfer (21) and methadone and switch (12 hits) or replacement (7 hits). In addition, available treatment guidelines and reviews were reviewed (Lintzeris et al., 2006; Crotty et al., 2020; Lintzeris et al., 2021).

### Current Strategies

There is no established algorithm for the transfer of patients from oral methadone to sublingual buprenorphine. The risk of buprenorphine-induced withdrawal basically depends on three parameters: dose of methadone, time interval between the exposure to methadone and buprenorphine, and level of physical dependence (Rosado et al., 2007). Most guidelines recommend a reduction of methadone to a low dose of about 30–40 mg(Lintzeris et al., 2006; American Society of Addiction, 2020; Lintzeris et al., 2021), and initiation of buprenorphine treatment after the first withdrawal symptoms have emerged, typically 24–48 h after the last dose of methadone, with an initial dose of 2–4 mg buprenorphine and additional 2–8 mg doses if needed to suppress withdrawal. In this case there is a minimal risk for precipitated withdrawal. A Clinical Opiate Withdrawal Scale [COWS, (Wesson and Ling, 2003)] score of 11–12 is indicative of a sufficient withdrawal to allow a safe and comfortable initiation onto buprenorphine (American Society of Addiction Medicine, 2020). The product license also suggests a reduction of methadone to 30 mg before switching the patient to buprenorphine after a minimum 24 h after the last methadone dose.

Generally the relative doses of methadone and buprenorphine and the time interval between the two are considered to be critical to avoid withdrawal symptoms. Lintzeris et al. (2021) in their recent systematic review identified 18 studies on transfer from methadone to buprenorphine, with an extreme variation on transfer protocols. Successful transfer was associated with lower pretransfer methadone dose (<60 mg).

In sum, a number of variables may contribute to outcome when switching a patient from methadone to buprenorphine (see Table 1). This review will focus on pharmacological aspects relevant in the transfer process—dosing issues, concomitant medications, and novel microdosing techniques.

**TABLE 1:** Transfer of patients from methadone to buprenorphien: relevant factors.

Severity of opioid dependence
Physical and mental condition
Length of methadone treatment
Methadone dose before transfer
Stable methadone dose or gradual reduction before transfer (fixed dose or flexible taper)
Waiting time between methadone and buprenorphine
Initial first-day dose of buprenorphine
Transfer duration (rapid or slowly)
Buprenorphine dose stabilization (final dose)
Management of withdrawal symptoms
Severity of opioid craving
Concomitant medications

### Dosing Issues—Switching from Low Dose and High Dose Methadone

Breen et al. (2003) studied different methadone transfer regimens in 51 outpatients at four clinics. Patients were maintained on their current methadone dose for 2 weeks. Patients on doses of 30 mg or more were randomly allocated to a fixed buprenorphine transfer at 30 mg methadone or a variable protocol (transfer when “uncomfortable”). The fixed dose protocol required patients to reduce their methadone dose by 2.5 mg per week to 30 mg for 1 week before being transferred to buprenorphine. In the variable dosing group patients reduced their dose by 2.5 mg per week until they reported withdrawal discomfort. A third group (transfer below 30 mg) were not randomized and transferred to burprenorphine from their entry dose. After at least 24 h 4 mg buprenorphine was given. Additional doses of buprenorphine were administered in the afternoon for the first 3 days if required (Induction regime: day 1: possible daily dose 4–8 mg, day 2: 0–16 mg, day 3: 0–24 mg, day 4 and 5. 0–24 mg buprenorphine). Clinical and withdrawal symptoms were assessed by various scales including the Subjective Opiate Withdrawal Scale and the Objective Opiate Withdrawal Scale (Handelsman et al., 1987). There were no differences between the first two groups, and—as expected—patients with doses less than 30 mg reported less discomfort than others. All but one patient stabilized on buprenorphine, and 38 of the 51 completed buprenorphine withdrawal.

Data from a retrospective case series of 25 patients (Salsitz et al., 2010) showed a low to moderate association between methadone and buprenorphine maintenance doses in patients transferred from stable methadone treatment to buprenorphine.

Some case series suggest that a more rapid transition from methadone to buprenorphine is possible. Law et al. (1997) reported 13 cases of male methadone patients on 20–30 mg who were rapidly and successfully transferred to buprenorphine 4 mg 24–26 h after their last methadone dose.

The most important study on this issue has been performed by Lintzeris et al. (2018) who examined the transfer from methadone to buprenorphine in 33 patients with lose doses of methadone (<30 mg, *N* = 9), medium doses (30–50 mg, *N* = 9), and higher doses (>50 mg, *n* = 15), mostly in an inpatient setting. A total of 22 patients received buprenorphine doses of >8 mg buprenorphine on day 1, 14 patients recieved 16 mg or more on day 1. Most patients had stabilized their daily buprenorphine dose by the third day of buprenorphine dosing. There were no complications in the first two groups, and three high-dose transfers experienced precipitated withdrawal. A total of 7 of the 33 participants returned to methadone within 1 week of attempted transfer.

Successful replacement from methadone (average dose 44 mg) by buprenorphine (12 mg average dose) was reported even in pregnant women in whom opioid withdrawal has to be avoided (Johnson and Martin, 2018). In this study a standardized protocol using low dose buprenorphine doses to minimize withdrawal symptoms was used (2 mg dose of buprenorphine hourly as needed for the first 24 h). A total of 20 pregnant women maintained on an average dose of 44 mg/day were successfully transitioned to a mean dose of 12, 6 mg buprenorphine/day.

An experimental study was reported by Rosado et al. (2007) who studied the acute effects of sublingual buprenorphine/naloxone in individuals with a higher level of physical dependence (*N* = 16, maintained on 100 mg methadone a day!). This was a randomized, double blind, triple dummy, within subject study. Phase 1 of the study: Conditions were sublingual buprenorphine/naloxone (4/1, 8/2, 16/4, 32/8 mg), intramuscular naloxone (0.2 mg), oral methadone (100 mg), or placebo. Phase 2: Conditions were methadone, placebo, naloxone. 100% of the buprenorphine/naloxone dose that precipitated withdrawal in phase 1 (full dose), and 50% of this dose was administered twice in a session. Results: In brief, 6 subjects did not complete the study. Of the 10 completers 3 tolerated a maximum dose of 32/8 mg without evidence of precipitated opioid withdrawal. For the 7 completers of both phases, split doses generally produced less withdrawal compared to full doses. The authors concluded that there is a considerable subject variability in sensitivity to buprenorphine’s antagonistic effects and that low repeated doses of buprenorphine/naloxone may be an effective mechanism for safe transfer from high dose methadone to buprenorphine.

The “high dose” methadone transfer to buprenorphine has also been adressed in a case series of 39 outpatients [35–120 ml methadone, (Conroy and Hill, 2013)] who completed transfer to bupenorphine (dosing protocol: last intake of methadone 36–40 h before transfer, followed by 2/0.5 mg buprenorphine/naloxone given at 9:30 and 10:30, 2 × 2/0.5 mg at 1:30, 8/2 mg at 12:30 and—if required—same dose again at 12:30, so that a total buprenorphine dose of 16–24 mg was given within 4 h). Two patients failed to complete the transfer. This study did neither report a mean methadone dose nor individual doses before transfer which limits the value of these findings.

Another study adressing transfer from higher doses of methadone to buprenorphine was perfomed by Naumovski and Batey (2015). A total of 29 outpatients (stabilized on 42.5–140 mg methadone/day) were transferred to buprenorphine. Patients were encouraged to reduce methadone prior to transfer and reduction was carried out at a rate of 5 mg twice a week as tolerated. A broad range of medications—metoclopramide, paracetamol, ibuprofen, buscupan, loperamide, and diazepam (5 mg tabs)—were also provided. Buprenorphine was given as a test dose of 4–4 mg, then 4–8 mg maximum 12 mg/day 1 if the test dose was tolerated well, 16–24 mg on day 2, 24–32 mg on day 3 with completion of transfer at day 4. A total of 29 patients completed the transfer process. Average dose of methadone for patients was 86.8 mg before transfer and 61.2 mg at begin of transfer! Six patients failed to complete the transfer process.

Oretti (2015) reported 7 retrospective case reviews of patients on high doses of methadone (60–120 mg) who were transferred to buprenophine in an inpatient setting. Buprenorphine was given after the first withdrawal symptoms were apparent (initial dose 4 mg, maximum dose over a 24 h period 24–32 mg buprenorphine!). Of the 7 patients, 6 completed the replacement process.

Levin et al. (1997) studied transfer from methadone 60 mg to buprenorphine in inpatients and suggested a 7-day changeover with gradual reduction of methadone (60-40-30-30-0 mg, 4–8 mg buprenorphine) in 19 patients. There were 15 patients who were completers. The noncompleters complained about withdrawal symptoms which they could not tolerate. In the others the methadone taper and buprenorphine initiation were successful.

### Medications to Smoothen the Transfer

Lofexidine was examined as a possible medication to reduce withdrawal symptoms in patients on >30 mg methadone when transferred to buprenorphine. Glasper et al. (2005) studied 23 opioid dependent inpatients on methadone 30–70 mg who were transferred to buprenorphine 12–16 mg/day. Following the last methadone morning dose buprenorphine was given in doses increasing from 4 to 16 mg maximum. All but two patients completed transfer to methadone. Patients with higher methadone dose (50–70 mg) had more severe opioid withdawal symptoms and required higher doses of daily lofexidine. In general, transfer from methadone 30–70 mg to buprenorphine was found to be relatively uncomplicated and can be facilitated by lofexidine.

Another approach was suggested by a Swiss group (Hess et al., 2011). They enrolled 11 subjects on methadone doses of 70–100 mg and switched them to buprenorphine by using a transdermal buprenorphine patch (35mu-g, delivery for over 96 h) 12 h after the last dose. The first doses of buprenorphine 2 mg were given 48–60 h after the last methadone intake, followed by 8 mg as an oral dose 96, 102, and 109 h after baseline. Transition was successfully completed in 10 of 11 patients.

A further variant was proposed by Azar et al. (2018) who used a transdermal fentanyl patch (25 mccg/h every 3 days) as a “bridge” from methadone to buprenorphine/naloxone in a patient.

Cortina et al. (2017) also described the case of a psychiatric opioid-dependent patient with prologend QT interval who was successfully transferred from methadone up to 180 mg (!) to buprenorphine using a transdermal buprenorphine patch (20 mcg/h).

Opioid antagonists were also studied to facilitate transfer from full opioid agonists to buprenorphine. A “rapid transition” approach was suggested by Ward et al. (2019) who reported the case of a patient on methadone 65 mg who was given naltrexone, soon followed by buprenorphine induction. Phillips et al. (2019) published a case in which a naloxone-induced opioid withdrawal was performed to rapidly initiate buprenorphine treatment.

### Microdosing Techniques

An alternative strategy in replacing methadone with buprenorphine is a micro-induction to avoid withdrawal symptoms by gradually accumulating buprenorphine and replacing methadone at the mu opioid receptor (Wong et al., 2021).

Low doses of partial agonists such as buprenorphine may not precipitate withdawal (Strain et al., 1995) but to date there are few data on the microdosing strategies, although the database is rapidly expanding. Hämmig et al. (2016) had suggested use of microdoses for induction of buprenorphine treatment with overlapping full opioid use (“Bernese method”). The authors had presented two cases, one of these patients was treated with high doses of pharmaceutical heroin and methadone during induction. This method required 10 or more days to achieve a therapeutic buprenorphine dose but there are also reports suggesting a faster induction (Lee et al., 2020).

Klaire et al. (2019) reported two cases initially brought to the emergency department who were on hydromorphone i.v., and received buprenorphine over a 5-day period, starting with 0.25 or 0.5 mg buprenorphine to finally 16 resp 12 mg buprenorphine.

Another microdosing approach was performed by Terasaki et al. (2019). This group implemented a 1-week buprenorphine microdosing protocol and reported a case series of 3 inpatients. Buprenorphine was given and gradually titrated at doses of 0.5 mg on day up to 12 mg on day 8. Methadone (two patients were on methadone 40mg, one on 100mg before transfer to buprenorphine) was abruptly stopped. Using this method, patients could be successfully transferred to buprenorphine with minimal symptoms of opioid withdrawal.

In addition, Aquino De et al. (2020) and Stanciu et al. (2020) also published case reports on rapid transition from methadone 75 and 30 mg to buprenorphine using a micro-dosing protocol.

A further approach was proposed by Callan et al. (2020) who reported the case of an inpatient transition from methadone 70 mg to buprenorphine using a “hydromorphone bridge” (24–48 mg daily) over a 7-day period. Hydromorphone is also used in the upcoming study of Wong et al. (2021). In Canada slow release morphine was used for transferring patients on methadone to buprenorphine (Ghosh et al., 2019a). Finally, Crane et al. (2020) reported the case of a 62-year-old patient on chronic methadone 80 mg daily referred to an emergency department with opioid overdose. He received 0.4 mg IV naloxone twice, then a naloxone infusion at 0.06 mg/h was started and an IV buprenorphine microdosing induction was initiated without interruption of methadone treatment. Buprenorphine was supplied in single use 1-ml vials of 0.3 mg/ml buprenorphine. Within 4 days the transfer was completed.

Finally, Becker and Frank (Becker et al., 2020) reported a succesful 5-day microdosing buprenorphine transfer (0.5 mg twice daily on day 1, 2 × 1 mg on day 2, 3 × 1 mg on day 3, 2 × 3 mg on day 4 and 4 × 3 mg on day 5) in 6 inpatients with chronic pain treated with various full opioid agonists without opioid withdrawal symptoms. One patient resumed full opioid agonist because buprenorphine was not effective enough to control her pain.

A very recent review on microinduction of buprenorphine/naloxone identied 18 papers with 63 patients who were successfully transitioned using microdosing techniques, mostly case reports and series (Ahmed et al., 2021). A variety of dosing schemes were used, with initial doses of often 0.2–0.5 mg (Hämmig et al., 2016; Payler, 2016; Caulfield et al., 2020; Crane et al., 2020; Rozylo et al., 2020). The time frame for transition ranged from 3 to 112 (!) days. Most patients transitioned over a period of 4–8 days. Another recent systematic review on this issue was performed by Moe et al. (2021). The review included 19 case studies/series and one feasability study with 57 patients. Again, there was a broad variety of dosing and treatment regimens. Starting doses ranged from 0.03 to 1.0 mg, maintenance doses from 8 to 32 mg. All patients achieved the desired maintenance dose, few experienced precipitated withdrawal.

Recently, Wong et al. (2021) published the protocol of an open-label study parallel-group, randomized study comparing rapid (2 day) micro-induction with a defined titration scheme of buprenorphine plus hydromorphone and standard induction of buprenorphine/naloxone for treatment of opioid use disorder. Although this study does not primarily adress patients on methadone it will further elucidate the prospects of microinduction with buprenorphine.

### Switching Patients from Methadone to Depot Buprenorphine Formulations

In recent years three different long-acting buprenorphine formulations have been developed and in part introduced into clinical practice (Soyka, 2021), including the weekly or monthly given depot injection CAM 2038 [Buvidal, (Walsh et al., 2017)], another depot injection RBP 6000 [Sublocade, (Haight et al., 2019)] and a buprenorphine implant (Ling et al., 2010; Rosenthal et al., 2013; Rosenthal et al., 2016). While transfer from sublingual to depot buprenorphine is easy to do, direct transfer from methadone to depot buprenorphine is usually done by first introducing sublingual buprenorphine treatment and then switching the patient to a depot formulation. The optimal tactics for direct transfer from methadone to long-lasting buprenorphine formulations has not been defined. A recent case series of Soyka and Groß (2021) of patients with opioid use disorder in a custodial setting suggests that a rapid transfer from methadone, in part at high dosages, to depot buprenorphine via an initial 4 mg sublingual buprenorphine dose is possible. Microdosing techniques to introduce a patient to depot buprenorphine medication has been recently advocated also by Tay Wee Teck et al. (2021).

## Discussion

Treatment induction onto buprenorphine is critical for retention and many patients drop out of treatment in this early phase. For the transfer of opioid dependent patients from methadone to buprenorphine no accepted clinical algorithim has been established and there are surprisingly few clinical and experimental studies on this important question [see also (Ghosh et al., 2019b)], mostly clinical case series. A number of studies in recent years indicate a growing interest in this subject. Different techniques of transferring patients on methadone to buprenorphine have been proposed. For patients on higher doses of methadone the conventional method is tapering patients to 30–40 mg methadone or less before buprenorphine treatment is initiated although there are a number of findings now indicating that transfer also from higher doses of methadone is possible (Lintzeris et al., 2021). While there are few studies comparing the switch to buprenorphine in patients on a stable dose of methadone or with a gradual reduction of methadone until the first mild to moderate withdrawal symptoms emerge it appears to be good clinical standard to lower methadone medication as far as possible before transfer to buprenorphine. A number of publications and case reports suggest direct transfer from higher doses of methadone to buprenorphine is possible, at least in a supportive setting with sufficient monitoring of the patient and possibly use of other “bridge” medications to soften withdrawal symptoms. Usually buprenorphine starts with a low dose of 2 mg or so, although some clicinians also start with a higher dose. Concerning medications to smoothen transition to buprenorphine a gold standard has not been established yet (Lintzeris et al., 2021).

There are also some novel techniques including microinduction of buprenorphine (Payler, 2016; Crane et al., 2020), use of transdermal buprenorphine patch (Raheemullah and Lembke, 2019), or other “bridge” techniques (Fentanyl, hydromorphone, slow release morphine, or others), or the concomitant administration of lofexidine, analgesics, or psychotropic drugs to reduce withdrawal symptoms. The possible advantage of microdosing technique is to minimize the risk for precipitated withdrawal and to improve patient comfort. It is a rather simple and safe method. There also is a lower threshold to treatment and no need to reduce methadone doses and risk destabilization. A possible disadvantage is that a transfer based on microdosing techniques might take somehow longer compared to conventional transfer, depending on the chosen dosing regimen.

In clinical practice, transition concepts should be easy to do also on the outpatient level with minimal discomfort for the patient to avoid discontinuation of treatment. Although the reviewed database is very limited the most promising novel strategy seems to be microdosing of buprenorphine during methadone treatment and a titration to clinical doses within a week or so. This should be used predominantly in patients on higher doses of methadone who do not wish or cannot reduce methadone to a level of 30–40 mg before initiating buprenorphine treatment. Clearly more studies are necessary to develop the optimal tactics for transfering patients from higher doses of methadone to buprenorphine, if required.
